# P-291. Whole genome sequencing (WGS) demonstrates high relatedness between carbapenemase-producing *Klebsiella pneumoniae* isolates cultured from paired rectal swab and blood specimens among hospitalized patients in Greece

**DOI:** 10.1093/ofid/ofae631.494

**Published:** 2025-01-29

**Authors:** Ritu Banerjee, Kerryl Greenwood-Quaintance, Christina Koscianski, Jordan Starkey, Amanda Rodning, Robin Patel, Paraskevi Mantzana, Areti Tychala, Georgios Meletis, Zoe Sereni, Petros Trikoupis, Margarita Oikonomou, Elisavet Kousouli, Anna Roussou, Smaragdi Charami, Alexandra Rovatsou, Christina Louka, Vasiliki Mamali, Georgios Chrysos, Georgios Alexandros Baziotis, Sophia Vourli, Panagiota Christina Georgiou, Styliani Louka, Eleni Poulianou, Polyxeni Karakosta, Efthymia Protonotariou, Spyros Pournaras, Olympia Zarkotou, Lemonia Skoura

**Affiliations:** Vanderbilt University Medical Center, Nashville, TN; Mayo Clinic, rochester, MN; Mayo Clinic, rochester, MN; Mayo Clinic, rochester, MN; Mayo Clinic, rochester, MN; Mayo Clinic, rochester, MN; Ahepa University Hospital, Thessaloniki, Thessaloniki, Greece; AHEPA Hospital, Thessaloniki, Thessaloniki, Greece; AHEPA University Hospital, Medical School, Faculty of Health Sciences, Aristotle University of Thessaloniki, Thessaloniki, Greece., Thessaloniki, Thessaloniki, Greece; CLEO, AHEPA University Hospital, Thessaloniki, Thessaloniki, Greece; Cleo, AHEPA Hospital, Thessaloniki, Thessaloniki, Greece; CLEO, AHEPA University Hospital, Thessaloniki, Thessaloniki, Greece; Tzaneio General Hospital of Piraeus, Athens, Greece., Piraeus, Attiki, Greece; Tzaneio Hospital, Piraeus, Attiki, Greece; Tzaneio General Hospital of Piraeus, Piraeus, Attiki, Greece; Tzaneio Hospital, Piraeus, Attiki, Greece; Tzaneio General Hospital of Piraeus, Piraeus, Attiki, Greece; Tzaneio General Hospital of Piraeus, Piraeus, Attiki, Greece; Tzaneio General Hospital of Piraeus, Piraeus, Attiki, Greece; “Attikon” University General Hospital, Athens, Attiki, Greece; National and Kapodistrian University of Athens, Athens, Zakinthos, Greece; Attikon University General Hospital, Medical School, National and Kapodistrian University of Athens, Athens, Greece, Athens, Attiki, Greece; “Attikon” University General Hospital, Athens, Attiki, Greece; Attikon University Hospital, Athens, Attiki, Greece; Attikon University General Hospital, Medical School, National and Kapodistrian University of Athens, Athens, Greece, Athens, Attiki, Greece; AHEPA University Hospital, Medical School, Faculty of Health Sciences, Aristotle University of Thessaloniki, Thessaloniki, Greece., Thessaloniki, Thessaloniki, Greece; Attikon University General Hospital, Medical School, National and Kapodistrian University of Athens, Athens, Attiki, Greece; Tzaneio General Hospital of Piraeus, Piraeus, Attiki, Greece; AHEPA University Hospital, Medical School, Faculty of Health Sciences, Aristotle University of Thessaloniki, Thessaloniki, Greece., Thessaloniki, Thessaloniki, Greece

## Abstract

**Background:**

Intestinal colonization with carbapenem-resistant organisms (CRO) may be considered to inform empiric antibiotic selection for symptomatic infection. However, it is unclear how often gut colonizing CRE isolates are related to bloodstream isolates in hospitalized patients with bacteremia, especially in areas with high CRO prevalence.

Minimum spanning tree of isolate relatedness

**Figure:**
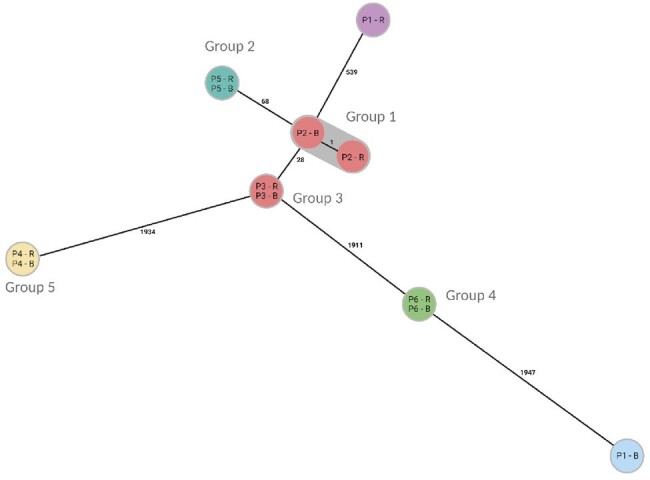

Isolates and corresponding patient number (P1-P6) and rectal (R) or blood (B) specimen are noted within circles and allelic differences are numerated on linking lines between the circles. Group 1 and 3 isolates are possibly related to each other. Created with BioRender.com.

**Methods:**

Patients with paired *K. pneumoniae* carbapenemase (KPC)-producing *K. pneumoniae* isolates cultured from surveillance rectal specimens and clinically collected blood specimens collected within 1-8 days of each other between 2019-2023 were identified at 3 Greek hospitals. Isolates were frozen and shipped to a reference laboratory in the U.S for whole genome sequencing (WGS) using Illumina MiSeq. Assembly and core genome multilocus sequence typing (cgMLST) analysis were performed with Ridom SeqSphere+ software. Isolate relatedness was based on the total number of allelic differences between each isolate: < 15, related; 16-50, possibly related; ≥ 51, unrelated.

**Results:**

One hundred eight paired isolates were identified from 54 unique patients; 6 pairs and 12 total isolates underwent cgMLST. In 5 of 6 pairs (83%), the rectal isolate was collected prior to the bloodstream isolate. Rectal and bloodstream isolates were related to one another in 5 of 6 (83%) pairs and were unrelated in 1 pair. Two pairs collected from 2 distinct patients admitted to the same intensive care unit (patients 2 and 3, Figure) were possibly related (16-50 allelic differences) to each other.

**Conclusion:**

In this pilot evaluation conducted in a setting of high CRO prevalence, KPC-producing *K. pneumoniae* isolated from paired rectal and blood specimens from individual patients were often related to each other, suggesting that intestinal colonization by this organism could inform empiric antibiotic treatment for bacteremia. Two patients from the same hospital unit had isolates that were possibly related, suggesting within hospital transmission. WGS is a powerful tool that can inform clinical and infection control practices. WGS of the remaining *K. pneumoniae* pairs and additional *Acinetobacter baumannii* pairs is ongoing.

**Disclosures:**

**Robin Patel, MD**, I already disclosed so please pull from disclosure for other abstracts: Advisor/Consultant|I already disclosed so please pull from disclosure for other abstracts: Grant/Research Support|I already disclosed so please pull from disclosure for other abstracts: Honoraria

